# To Sleeve or NOT to Sleeve in Bariatric Surgery?

**DOI:** 10.5402/2012/674042

**Published:** 2012-08-16

**Authors:** P. W. J. van Rutte, M. D. P. Luyer, I. H. J. T. de Hingh, S. W. Nienhuijs

**Affiliations:** Department of Surgery, Catharina Hospital Eindhoven, 5602 ZA Eindhoven, The Netherlands

## Abstract

Morbid obesity has become a global epidemic during the 20th century. Until now bariatric surgery is the only effective treatment for this disease leading to sustained weight loss and improvement of comorbidities. The sleeve gastrectomy is becoming a promising alternative for the gold standard the gastric bypass and it is gaining popularity as a stand-alone procedure. The effect of the laparoscopic sleeve gastrectomy is based on a restrictive mechanism, but a hormonal effect also seems to play an important role. Similar results are achieved in terms of excess weight loss and resolution of comorbidities compared to the gastric bypass. Inadequate weight loss or weight regain can be treated by revisional surgery. Complication rates after LSG appear to be lower compared with gastric bypass. General guidelines recommend bariatric surgery between the age of 18 and 65. However bariatric surgery in the elderly seems safe with respect to weight loss and resolution of comorbidities. At the same time weight loss surgery is more often performed in adolescent patients failing weight loss attempts. Even though more studies are needed describing long-term effects, there is already enough evidence that this technique is an effective single procedure for a considerable proportion of obese patients.

## 1. Introduction

Obesity is one of the most upcoming health issues these days as it has become a global epidemic during the 20th century, affecting 10–30% of the adult population in Europe according to the latest reports of the WHO. Obesity is responsible for 2–8% of health costs and 10–13% of deaths are related to this disease. Obesity is not only prevalent in adults, but is also increasing amongst children. Children with overweight are prone to be overweight in early adulthood with the risk of developing cardiovascular disease, type 2 diabetes, and orthopedic problems as well as mental disorders, underachievement in school, and lower self-esteem. Therefore, treatment to control obesity is essential. Until now, bariatric surgery is the only effective treatment for morbid obesity as it leads to sustained weight loss and improvement of comorbidities [1]. Besides a reduction in excess weight, bariatric surgery also improves metabolic changes, for example, type 2 diabetes and hypercholesterolemia and organ functioning such as sleep apnea and hypertension, thereby effectively decreasing obesity-related morbidity and mortality [2–4]. In the Swedish obese subjects trial (SOS) surgically treated subjects (*n* = 2010) were compared with medically treated patients (*n* = 2037). This study showed that surgical intervention leads to significantly more weight loss after 2 and 10 years compared to medical treatment [4]. This is in line with other studies showing that surgery results in more excess weight loss than conservative treatment [3, 5]. A meta-analysis performed by Buchwald et al. even showed 61.2% excess weight loss after surgical intervention [6]. Furthermore, bariatric surgery leads to a sustained improvement of comorbidities for more than 5 years in most patients and lowered costs of medication [7, 8]. Patients who have had surgical treatment even show notable improvement in quality of life and psychiatric dysfunction after 2-year follow-up, compared to conservatively treated patients [5, 9]. Bariatric surgery significantly decreases the mortality rate in obese subjects [10]. However a recent study by Plecka et al. comparing morbidity and mortality in the morbid obese with the general population concluded that the risk of death remains increased after bariatric surgery in morbid obese patients [11].

Above mentioned advantages were achieved with various surgical techniques. The gastric bypass procedure is accepted as the gold standard. The main alternative used to be an adjustable band however, the high revision rate has dramatically decreased usage of this technique, especially in Europe. Nowadays the alternative is a sleeve gastrectomy [12, 13]. This technique has similar results in terms of weight loss and improvement of comorbidities compared to the gold standard, the gastric bypass, and seems promising for the future [14].

## 2. What Is a Sleeve? 

The laparoscopic sleeve gastrectomy (LSG) is an evolving surgical technique to treat morbid obesity in which the fundus of the stomach is resected. Initial publications about the LSG concerned the super obese (BMI > 60 kg/m^2^) and the high-risk patients with many comorbidities that underwent an LSG as a staged procedure. In these patients the sleeve gastrectomy was used as a first stage intervention in order to reduce morbidity and mortality and to lose weight to facilitate a second stage operation, such as a gastric bypass or a biliopancreatic diversion after at least six months [15, 16]. However, since the beginning of this century the sleeve gastrectomy has started to gain more popularity as a primary bariatric intervention as this technique is technically easier and relatively faster than other bariatric procedures, showing a low complication rate, a mean excess weight loss comparable with the other surgical techniques, and significant reduction in comorbidities [16, 17]. Nowadays many surgeons use the LSG within their standard bariatric procedures because of these advantages. 

## 3. How to Sleeve? 

During the laparoscopic procedure, a tubular gastric pouch of 75–120 mL is created by inserting a bougie along the lesser curvature of the stomach. As this treatment modality is fully evolving as a stand-alone procedure still no consensus has been reached what size bougie should be ideally used, where to start stapling, and where to end. Usage of 32 French up to 60 French bougies has been reported. However the smaller bougie sizes of 32–42 French show better results regarding excess weight loss and weight regain [18–20]. The pouch is created using a stapler starting proximal to the pylorus in order to preserve the antral pump and continuing parallel to the lesser curvature of the stomach to the angle of His ending approximately 1 cm to the left of the esophagus. Another point of discussion is the care of the staple line in order to prevent staple line leakage. The literature describes three methods, namely, oversewing the staple line, buttressing the staple line with absorbable material, or no staple line reinforcement. Various reports describe that reinforcing the staple line by a buttress, or by suturing the staple line, would decrease the risk of hemorrhage and leakage, but there is no evidence about the best method [21, 22]. Although still no unambiguous surgical technique exists we have come to the general agreement that creating a gastric remnant between 75 and 120 mL results in optimal excess weight loss and during our bariatric procedures we create the sleeve using a 34 French bougie and start stapling about 5 centimeters proximal to the pylorus. 

## 4. Why to Sleeve? 

Weight loss after sleeve gastrectomy is partly achieved by the restrictive mechanism, as the tubular stomach is small and merely resistant to stretching after resection of the gastric fundus. Additionally decreased levels are found of ghrelin, which is secreted by the oxyntic cells of the gastric fundus in response to fasting. Also elevated levels are found of the hormones peptide-YY (PYY), produced mainly in the ileum and the colon, and glucagon-like peptide-1 (GLP-1), secreted in the enteroendocrine L cells in the intestines. Ghrelin levels rise during the period of fasting, reaching a peak just before consumption of a meal. This enzyme stimulates appetite. During eating the release of PYY and GLP-1 increases, reaching a peak level 1 to 2 hours after food consumption, influenced by the amount of calories that are ingested. The hormone PYY decreases appetite. Glucagon-like peptide enhances the insulin production and it's release from the pancreas. By eliminating the gastric fundus during the sleeve gastrectomy, secretion of ghrelin is abolished, causing loss of appetite. Various studies show higher levels of PYY and GLP-1 after sleeve gastrectomy, leading to extended satiety a decrease in gluconeogenesis, and increase in insulin secretion, respectively [2, 7, 23]. Thus, the effect of the LSG is based on a restrictive mechanism and a hormonal effect seems to play an important role. 

At the Catharina Hospital Eindhoven we have been performing LSG as a primary treatment modality for 6 years with acceptable results. Between August 2006 and April 2011 686 sleeve gastrectomies have been done at our surgical department. In this patient population a median excess weight loss of 69% (10%–200%) was seen after a median followup of 18 months (7–68 months). Comorbidities improved in 75% of the cases. Revisional surgery was performed in 8.9% of the patients. In all of them the gastric sleeve was converted to a Roux-en-Y gastric bypass. After revision these patients had a significant additional excess weight loss and a further decrease in comorbidities. 

Several studies reporting large series have shown that LSG is safe and effective in terms of weight loss and improvement of comorbidities in the first postoperative years. A recent study reporting a large series of 1000 LSG even found an excess weight loss of 86.6% in the first postoperative year, 84.2% after two years, and 84.5% after 3 years [18]. A systematic review by Brethauer et al. showed a mean excess weight loss (%EWL) of 55.4% (33–85%; *n* = 1662), and the mean BMI decreased from 51.2 kg/m^2^ to 37.1 kg/m^2^ after SG (*n* = 1940) [24]. Other studies showed %EWL of 60–84% in the first postoperative year [19, 25]. The mean excess weight loss after LSG is equal to Roux-en-Y gastric bypass in the first two postoperative years [26]. Pre-existent comorbidities appear to improve or even resolve after a laparoscopic sleeve gastrectomy. Resolution of type-II diabetes occurs in 60–96% of the patients. This process starts even before losing weight as levels of ghrelin decrease directly after surgery. Similar resolution of diabetes is seen after RYGB (84%), induced by the same mechanism [27, 28]. Other comorbidities as hypertension, dyslipidemia, arthritis, and sleep apnea improve significantly after surgery, but no statistically significant difference is found between LSG and RYGB [29–31]. Laparoscopic sleeve gastrectomy would also cause less nutrient deficiencies after surgery than RYGB [32]. Various complications are reported after bariatric surgery. Complication rates after LSG vary in the literature ranging from 2.9 to 9.5%, which appear to be lower than the amount of complications seen after RYGB, varying between 4.6 and 20.5% in the literature [2, 24, 33, 34]. Most concerns minor complications such as wound infection or mild bleeding. A major complication is staple line leakage, occurring in 1.7–2.4% after the sleeve gastrectomy, according to Aurora et al. [30, 35]. Anastomotic leakage after RYGB occurs in 0.6–2.1% of the cases [2]. Another frequently reported comorbidity associated with obesity is gastroesophageal reflux disease (GERD) [36]. It is already known that Roux-en-Y gastric bypass alleviates GERD [37]. However the literature shows no consensus about the effect of LSG on GERD [38]. Patients with persisting or newly developed GERD after LSG can be treated with a conversion to RYGB [39]. Mortality rates after LSG vary between 0.1 and 0.3% [19]. Finally, sustainability is an important quality of bariatric surgery. Although no consensus exists choosing the right size and sleeve volume in LSG, a removed gastric volume of less than 500 mL seems to be a predictor for weight regain [40]. Only few studies describe long-term weight effect on weight loss and comorbidities after LSG. However, present studies show %EWL of 65–77.5% and 50–53% three years and six years after LSG, respectively [19, 25]. Inadequate weight loss or weight regain can be treated by revisional surgery. Re-sleeve gastrectomy is feasible in case of dilated initial sleeve, but carries higher risk of postoperative complications [41, 42]. An alternative treatment for insufficient weight loss is conversion to RYGB [39].

## 5. When to Sleeve?

The laparoscopic sleeve gastrectomy still is a well-accepted treatment modality as a first stage procedure in the super-obese patient or the high-risk patient with multiple comorbidities. Various studies showed a significant decrease in comorbidities and medication use after first stage LSG [24]. Although RYGB still is the Gold Standard LSG can serve as a good alternative given the good results reported after laparoscopic sleeve gastrectomy as a primary treatment modality. In case of volume eaters, LSG is a preferable option as the restrictive mechanism limits the amount of ingested food. LSG is not feasible in patients with GERD as it might worsen after the procedures [38]. General guidelines recommend bariatric surgery in obese patients between the age of 18 and 65. Several studies show that bariatric surgery in the elderly patient is safe with respect to weight loss and resolution of comorbidities [43–45]. A recent study concluded that sleeve gastrectomy is equally effective in patients older than 60 years compared to the younger obese population in terms of weight loss and resolution of comorbidities, but more care must be taken considering vitamin and protein deficiencies as they arise more frequently in the elderly obese postoperatively [46].

With the dramatic decrease in the age of onset of obesity, weight loss surgery is more often performed in adolescent patients who have failed weight loss attempts [47]. Laparoscopic sleeve gastrectomy may serve as a good option in this population as it has both a restrictive and a hormonal effect and it can be revised relatively easily. Still no long-term results have been reported regarding bariatric surgery in obese adolescents.

## 6. Conclusion

The laparoscopic sleeve gastrectomy is gaining popularity as primary treatment for obesity. As this relatively new procedure is still in evolution, still there is no uniform technique. It has similar results in terms of weight loss and improvement of comorbidities compared to the gold standard, the gastric bypass. Even though more studies are needed describing long-term effects, there is already enough evidence that this technique is the effective single procedure for a considerable proportion of obese patients. If insufficient weight loss or weight regain is encountered, likewise all other procedures, revision of a sleeve into a bypass seems more feasible than the limited options after a bypass as first procedure. A laparoscopic sleeve gastrectomy is therefore a viable option for all obese patients, except for those with pre-existent GERD.
